# Magnetoencephalographic evaluation of repaired lip sensation in patients with cleft lip

**DOI:** 10.1371/journal.pone.0274405

**Published:** 2022-09-22

**Authors:** Chihiro Kitayama, Eriya Shimada, Hiroki Hihara, Akitake Kanno, Nobukazu Nakasato, Yoshimichi Imai, Akimitsu Sato, Ryuta Kawashima, Kaoru Igarashi, Hiroyasu Kanetaka

**Affiliations:** 1 Division of Craniofacial Anomalies, Tohoku University Graduate School of Dentistry, Sendai, Miyagi, Japan; 2 Department of Orthodontics and Speech Therapy for Craniofacial Anomalies, Tohoku University Hospital, Sendai, Miyagi, Japan; 3 Division of Advanced Prosthetic Dentistry, Tohoku University Graduate School of Dentistry, Sendai, Miyagi, Japan; 4 Department of Epileptology, Tohoku University Graduate School of Medicine, Sendai, Miyagi, Japan; 5 Department of Electromagnetic Neurophysiology (Ricoh), Tohoku University, Sendai, Miyagi, Japan; 6 Department of Plastic and Reconstructive Surgery, Tohoku University Graduate School of Medicine, Sendai, Miyagi, Japan; 7 Department of Functional Brain Imaging, Institute of Development, Aging and Cancer, Tohoku University, Sendai, Miyagi, Japan; 8 Division for Interdisciplinary Integration, Liaison Center for Innovative Dentistry, Tohoku University Graduate School of Dentistry, Sendai, Miyagi, Japan; Thamar University, Faculty of Dentistry, YEMEN

## Abstract

**Background:**

Cleft lip is the most common congenital anomaly worldwide. Nevertheless, lip somatosensory characteristics of patients with cleft lip after cheiloplasty have not yet been determined. The present study used magnetoencephalography to objectively evaluate the lip sensation in patients with unilateral cleft lip to establish a new objective evaluation method.

**Methods:**

Participants were 15 patients with unilateral cleft lip after cheiloplasty (UCL group), and 30 healthy young subjects (control group). Five points of the upper and lower lips were stimulated electrically to measure somatosensory evoked magnetic fields (SEFs). The sources of the magnetic fields were modeled as single equivalent current dipoles (ECDs). ECDs located on the central sulcus by superimposition on magnetic resonance images were analyzed. Latency and intensity at 50–75 ms (cP60m) observed in the UCL group were compared with those in the control group. Thresholds of tactile stimuli in both groups were obtained using Semmes–Weinstein monofilaments for subjective sensory evaluation.

**Results:**

No significant difference was found in the intensity of the cP60m or subjective evaluation between the groups. However, the latency of the cP60m was significantly longer in the upper lip of the UCL group than in the control group.

**Conclusions:**

SEFs showed a difference in lip sensation between the UCL group and the control group, suggesting that longer latency might be caused by the effects of surgical scarring on the neurotransmission pathway. These results suggest SEFs as useful for the objective evaluation of lip sensations. This study might improve future surgical procedures and lip functions of patients with cleft lip.

## Introduction

Cleft lip and/or cleft palate (CL/P) are maxillofacial abnormalities which occur in approximately one in 700 infants [1, 2]. Treatment protocols for CL/P differ among institutions, but cheiloplasty (cleft lip repair) is usually performed between 3 and 6 months after birth [1–3]. Historically, cheiloplasty methods involved a linear incision such as the Miraut method [4]. Subsequently, the valvular incision method used for the Hagedorn method was introduced, with subsequent square flap procedures used, such as the Le Mesurier method [5], and triangle flap procedures such as the Tennison method [6]. Rotation advancement flap procedures such as the Millard method [7] have recently been applied widely. Morphological improvement has progressed considerably [4].

Nevertheless, the lip somatosensory characteristics of patients with CL/P after cheiloplasty remain unclear. Perioral somatosensation, especially of the lips, greatly affects articulation, mastication, and swallowing functions [8, 9]. Trotman et al. reported that abnormalities in lip morphology and function of patients with CL/P are attributable to impaired sensorimotor integration [10]. Furthermore, Chapparo et al. reported that sensory integrative dysfunction in patients with CL/P is associated with learning, language, and behavioral problems [11]. Normal sensory function is necessary for normal perioral motor functions and development [12, 13]. Comparisons of the two-point threshold and the cold and warm thresholds revealed no significant difference between patients with CL/P and healthy subjects [14, 15]. However, one report has described that patients with CL/P have abnormal sensations [16]. Therefore, subjective methods might not definitively determine the oral somatic sensations. Previous evaluations of oral somatic sensations have depended mostly on subjective evaluations by patients because objective evaluation is difficult [17].

Human lips include numerous peripheral receptors [18] and have a larger cortical representation than other areas in the somatosensory homunculus [19]. Upper lip receptors are site-specific and encode accurate somatosensory information [20]. Therefore, methods for imaging the human brain might be suitable for assessing lip sensation. However, few earlier studies of patients with CL/P have adopted brain imaging methods [21, 22]. Shinagawa et al. reported the effects of differences in motor function and somatosensory for widespread activation of the primary sensorimotor cortex in patients with CL/P when pronouncing "pa", but they presented no conclusions because of interindividual variations and a small sample size [21]. Nevalainen et al. used MEG to examine changes in lip sensation for oral plate therapy in children, including patients with CL/P, but described no findings about changes in lip sensation caused by cleft lip repair [22]. Consequently, although earlier reports show lip somatosensory effects on the temporomandibular joint, mastication, pronunciation, as well as learning disabilities and development. Objective evaluation is poor at present. Therefore, lip somatosensation must be evaluated objectively in patients with cleft lip (CL).

This study used magnetoencephalography (MEG) for objective evaluation of the somatosensory evoked magnetic fields (SEFs) associated with oral somatic sensations of patients with CL. MEG has higher temporal resolution than electroencephalography, and higher spatial resolution than functional magnetic response imaging and near-infrared spectroscopy [23]. MEG can visualize and can facilitate quantitative evaluation of the sensory signal source. Currently, MEG is used widely for detailed measurement of brain functions related to somatic sensations in clinical and research situations. Although MEG has been used to measure SEFs of the oral area to investigate somatotopic organization [20, 24–33], SEFs of the lips in patients with CL have not been evaluated yet. This study investigated the effects of surgical treatment on lip sensations by comparing SEFs induced by stimulation to repaired lips in patients with CL and normal lips in healthy subjects. In addition, subjective sensations using Semmes–Weinstein monofilaments were measured for comparison.

The working hypotheses are the following:

1. The latency of the SEFs is prolonged because of impaired neurotransmission caused by surgery.
2. The intensity of the SEFs is decreased because of reduced neural density around the surgical scar.

For this study, we establish the objective evaluation method using MEG for patients with CL and provide lip sensation data of patients with CL.

## Materials and methods

### Subjects

The patient group consisted of 15 patients with unilateral CL (UCL group) (7 male, 8 female; aged 15.0–29.0 years, mean 19.1 years): 9 with CL on the left and 6 with CL on the right. Of them, 13 patients were treated with cheiloplasty at Tohoku University Hospital using the modified Millard method at age 0.24–0.62 years, mean 0.36 years; 2 patients were treated with cheiloplasty (surgical methods unknown) at other hospitals at 3 months after birth. Table 1 presents the number of patients treated using each surgical method following cheiloplasty in the UCL group. The inclusion criteria of the UCL group were patients who had unilateral CL/P and who had received cheiloplasty among the patients of Department of Orthodontics and Speech Therapy for Craniofacial Anomalies, Tohoku University Hospital. The exclusion criteria of the UCL group were (1) persons who had not received cheiloplasty, (2) persons with other craniofacial anomaly, (3) persons with oral mucosal diseases, (4) persons with a severe vomiting reflex, (5) persons who were ineligible for magnetic resonance (MR) image examination, (6) persons who were judged to be left-handed according to the Edinburgh handedness test [34], (7) persons with nervous system diseases, and (8) persons who were judged by the principal investigator or the research coordinator to have difficulty participating in the research.

**Table 1:** Numbers of patients treated with surgical methods following cheiloplasty in the UCL group.

	Cheiloplasty	Palatoplasty	Alveolar bone grafting	Orthodontic treatment	Orthognathic surgery	Lip revision surgery
Number of patients	15	13	14	10	3	7

The control group consisted of 30 healthy young subjects (19 men and 11 women; 19.6–30.0 years, mean 24.4 years). The inclusion criteria of the control group were persons who have no cleft lip or palate or other craniofacial anomaly. The exclusion criteria of the control group were the same as those of the UCL group, excepting (1). In addition, the exclusion criteria of the control group included (9) persons who had received surgery around the oral cavity.

### Tactile stimulation threshold

Subjective evaluation of the threshold of the tactile sense was done using Semmes–Weinstein monofilaments (Sakai Medical Co., Ltd., Tokyo, Japan). The target force was set in the device for filaments numbered as Nos. 1.65, 2.36, 2.44, 2.83, 3.22, 3.61, 3.84, and 4.08, respectively using target forces of 0.008 g, 0.02 g, 0.04 g, 0.07 g, 0.16 g, 0.40 g, 0.60 g, and 1.00 g. An experienced experimenter applied one filament perpendicular to the lip surface, pressed for 1 s, maintained the force for 2 s as the filament bent slightly, then returned it to the original position for 1 s. During the test, the supine subject was instructed to raise the right hand if a stimulus was felt. Stimulation was continued from the strongest filament to the weakest filament in order. The first unrecognized stimulation was regarded as the lower limit threshold. Next, the stimulation series was performed from the filament at the lower limit threshold to the strongest filament in order. The first recognized stimulation was regarded as the upper limit threshold. After this experiment was repeated three times, the mean of the maximum value of the lower limit threshold and the minimum value of the upper threshold was adopted as the tactile threshold [35].

### MEG recordings

**Fig 1:**
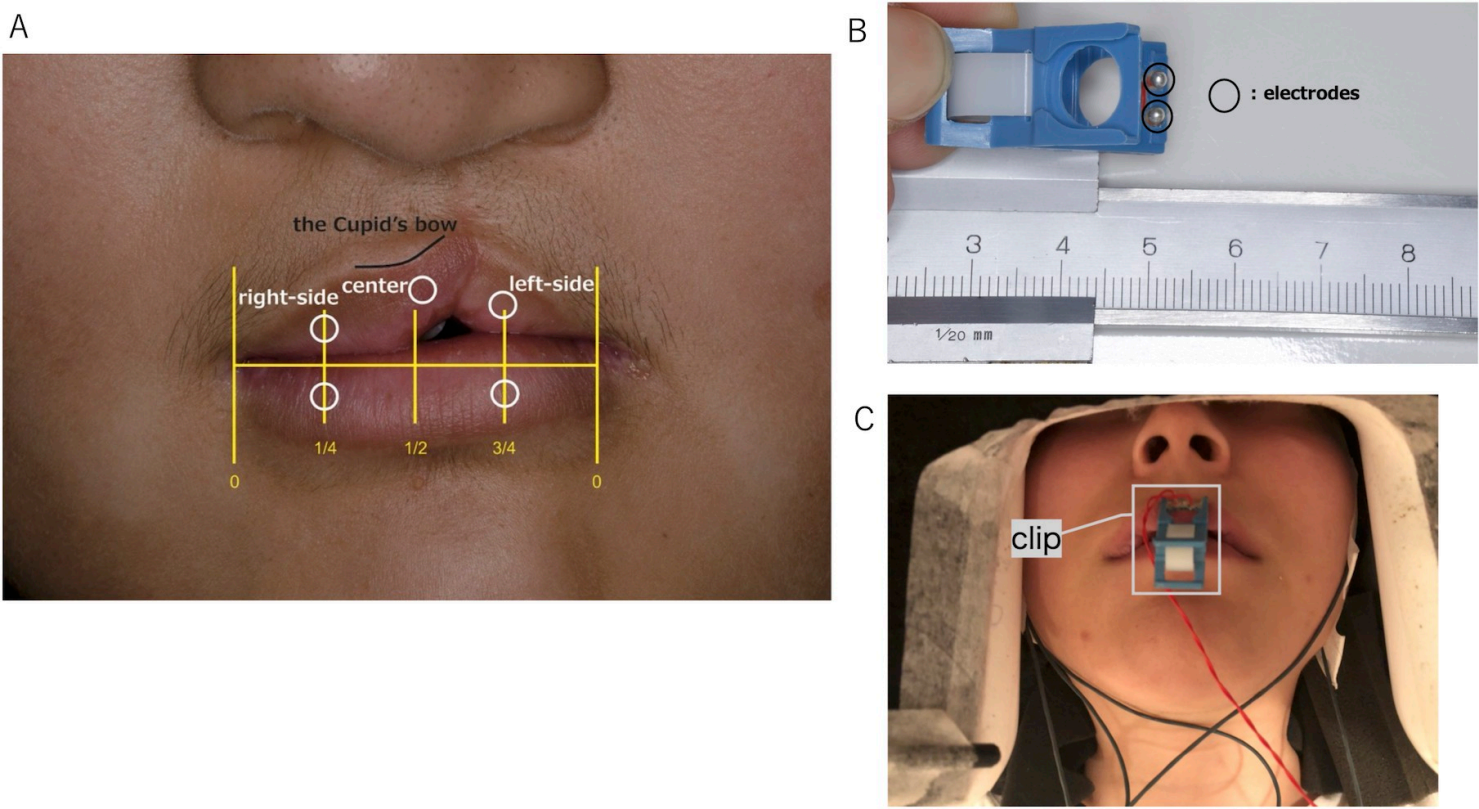
Stimulation points of the lips and stimulation device. (A) Lips of a patient with cleft lip. Stimulation points were at three locations on the upper lip (right side, center, and left side) and two on the lower lip (right side and left side). Stimulation points on the right and left sides were decided by measuring the distance between the right and left corners of mouth. The right and left stimulation points were at 1/4 of the distance from the right corner and at 3/4 of the distance from the right corner, respectively. The center stimulation point of upper lip was at the center of Cupid’s bow. Circles show the stimulation points. (B) Clip-type stimulation device with electrodes. (C) Stimulation device positioned on the center of the upper lip.

For all subjects, SEFs induced by electrical stimuli to three points of the upper lip (right side, center, and left side) and two points of the lower lip (right side and left side) were measured. The right and left stimulation points were determined based on the distance between the right and left corners of the mouth. The point at 1/4 of the distance from the right corner was set as the right stimulation point. The point at 3/4 was set as the left stimulation point. The stimulation point of the upper lip center was set at the center of Cupid’s bow (Fig 1A). We used a handmade clip with silver-ball electrodes (Unique Medical Co., Ltd., Tokyo, Japan) [33] to apply electrical stimuli to the lips (Fig 1B and 1C). The electrical stimuli consisted of constant current biphasic pulses with 0.2 ms duration delivered at 0.7 Hz. The sensory threshold was found using the method of limits. Intensity five times that of the threshold was applied. Because the stimulation strength was lower than the pain threshold, no subject felt any pain.

The SEFs were measured using a whole-head 200-channel MEG system (PQA160C; Ricoh Co., Ltd.) in a magnetically shielded room. The subject lay supine, with head positioning determined by the positions of five fiduciary markers consisting of induction coils placed at known locations on the scalp. The head shape and coil positions were established using a three-dimensional digitizer (FastSCAN Cobra; Polhemus Inc., Colchester, VT) based on three-dimensional MR images using a 3T MR system (Achieva; Philips Healthcare, Best, the Netherlands). The MEG signals, which were recorded from 50 ms before to 300 ms after the trigger point, were filtered from 0.5 to 1000 Hz, and were digitized at 2000 Hz. Data for about 150 stimuli were averaged.

Activities in the hemisphere contralateral to the stimulated side were analyzed, or activities of both hemispheres were combined for stimuli applied to the upper lip center. We analyzed the activities with peak latency around 50 ms to 75 ms with posterior orientation (cP60m) according to our earlier study [27]. The sources of the magnetic fields were modeled as single equivalent current dipoles (ECDs). The location and moment of source were estimated in a spherical conductor model (source of modeling software: MEG laboratory) based on Sarvas’ law [36]. Superimposition of ECDs on MR images showed that all ECDs were located on the central sulcus. Subsequently, we identified localization of the primary somatic sensation field in reference to localization of the left wrist activity. The goodness-of-fit values of the chosen dipoles exceeded 80%.

### Statistical analysis

The right and left sides of the upper lips of patients in the UCL group were grouped into the non-cleft side (left in 6 patients, right in 9 patients) and the cleft side (left in 9 patients, right in 6 patients). Values on these sides were compared with the average values for the upper right and left sides of lips of the control group. The average values of the right and left hemispheres on the center of the upper lip were compared between the groups. Statistically, the tactile threshold, the stimulation intensity and latency were compared between groups using multiple linear regression analysis with age and sex assumed as independent variables. Differences for which *p* < 0.05 were inferred as significant.

### Ethics

This study was approved by the Ethics Committee of Tohoku University Graduate School of Dentistry (protocol number: 2018-3-015) and was performed according to the Declaration of Helsinki. We obtained written informed consent from all participants, and from a guardian for cases in where the patient was a minor.

## Results

### Tactile stimulation threshold

**Fig 2:**
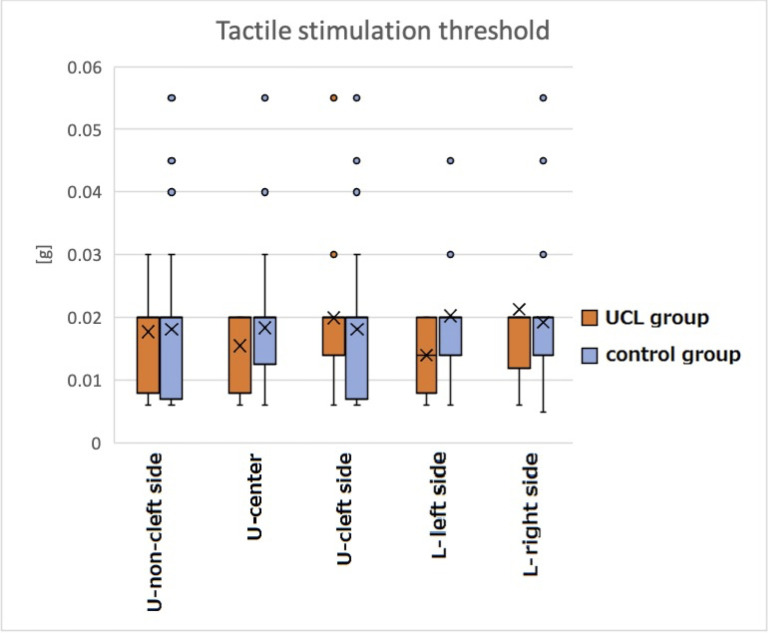
Tactile stimulation threshold (g) measured using the Semmes–Weinstein monofilament device for subjective evaluation. No significant difference was found for any measuring point between the groups: U, upper; L, lower.

Thresholds of tactile stimuli (mean ± standard deviation [SD]) in the UCL group were 0.018 ± 0.009 g, 0.015 ± 0.006 g, 0.020 ± 0.011 g, 0.014 ± 0.006 g, and 0.021 ± 0.023 g, respectively, on the non-cleft side, center, cleft side, lower left side, and lower right side. Values in the control group were 0.018 ± 0.012 g, 0.018 ± 0.011 g, 0.020 ± 0.010 g, and 0.018 ± 0.011 g, respectively, on the upper left and right sides (combined), center, lower left side, and lower right side. No significant difference was found between the UCL group and the control group (Fig 2). Table 2 presents results for tactile stimulation thresholds obtained using multiple linear regression analysis.

**Table 2:** Results for tactile stimulation thresholds from multiple linear regression analysis.

	Coefficient	95% conf. interval		*p*-value
U-non-cleft side	0.0022	-0.0071	0.1153	0.639
U-center	-0.0026	-0.0111	0.0060	0.550
U-cleft side	0.0000	-0.0089	0.0089	0.997
L-left side	-0.0063	-0.0142	0.0017	0.121
L-right side	-0.0025	-0.0173	0.0123	0.733

Age and sex adjusted. U, upper; L, lower.

### Latency of SEFs

**Fig 3:**
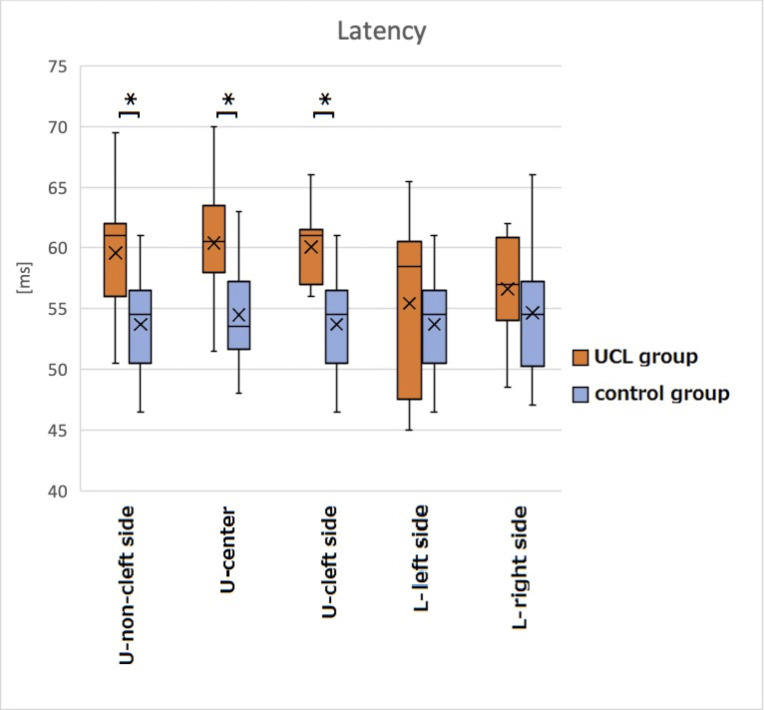
Latency of the cP60m (ms). Latencies of the cP60m in the UCL group were significantly longer on the non-cleft side, center, and cleft side of the upper lip than those in the control group (*p < 0.05): U, upper; L, lower.

Latencies of the cP60m (mean ± SD) in the UCL group were 59.955 ± 4.826 ms, 60.412 ± 4.599 ms, 60.091 ± 3.175 ms, 55.444 ± 6.755 ms, and 56.625 ± 4.121 ms, respectively, on the non-cleft side, center, cleft side, lower left side, and lower right side. Values in the control group were 53.689 ± 3.891 ms, 54.500 ± 4.052 ms, 54.306 ± 4.882 ms, and 54.643 ± 5.113 ms, respectively, on the upper left and right sides (combined), center, lower left side, and lower right side. Latencies of the cP60m in the UCL group were significantly longer on the non-cleft side, center, and cleft side of the upper lip than in the control group (*p* < 0.05) (Fig 3). Table 3 presents results obtained for latency of SEFs using multiple linear regression analysis.

**Table 3:** Results obtained for latency of SEFs using multiple linear regression analysis.

	Coefficient	95% conf. interval		*p*-value
U-non-cleft side	6.903	3.371	10.436	*0.0001
U-center	3.687	0.042	7.332	*0.048
U-cleft side	6.178	2.077	10.278	*0.004
L-left side	2.336	-4.179	8.850	0.467
L-right side	3.318	-2.423	9.059	0.246

Age and sex adjusted. U, upper; L, lower

*p < 0.05.

### Intensity of SEFs

**Fig 4:**
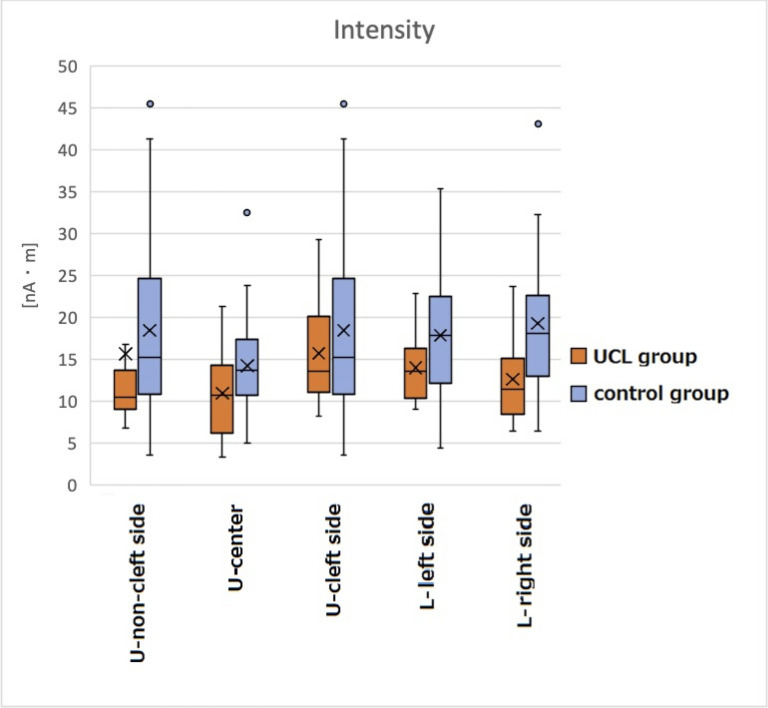
Intensity of the cP60 (nA•m). Signal intensity of the cP60m indicated no significant difference between the groups: U, upper; L, lower.

**Fig 5:**
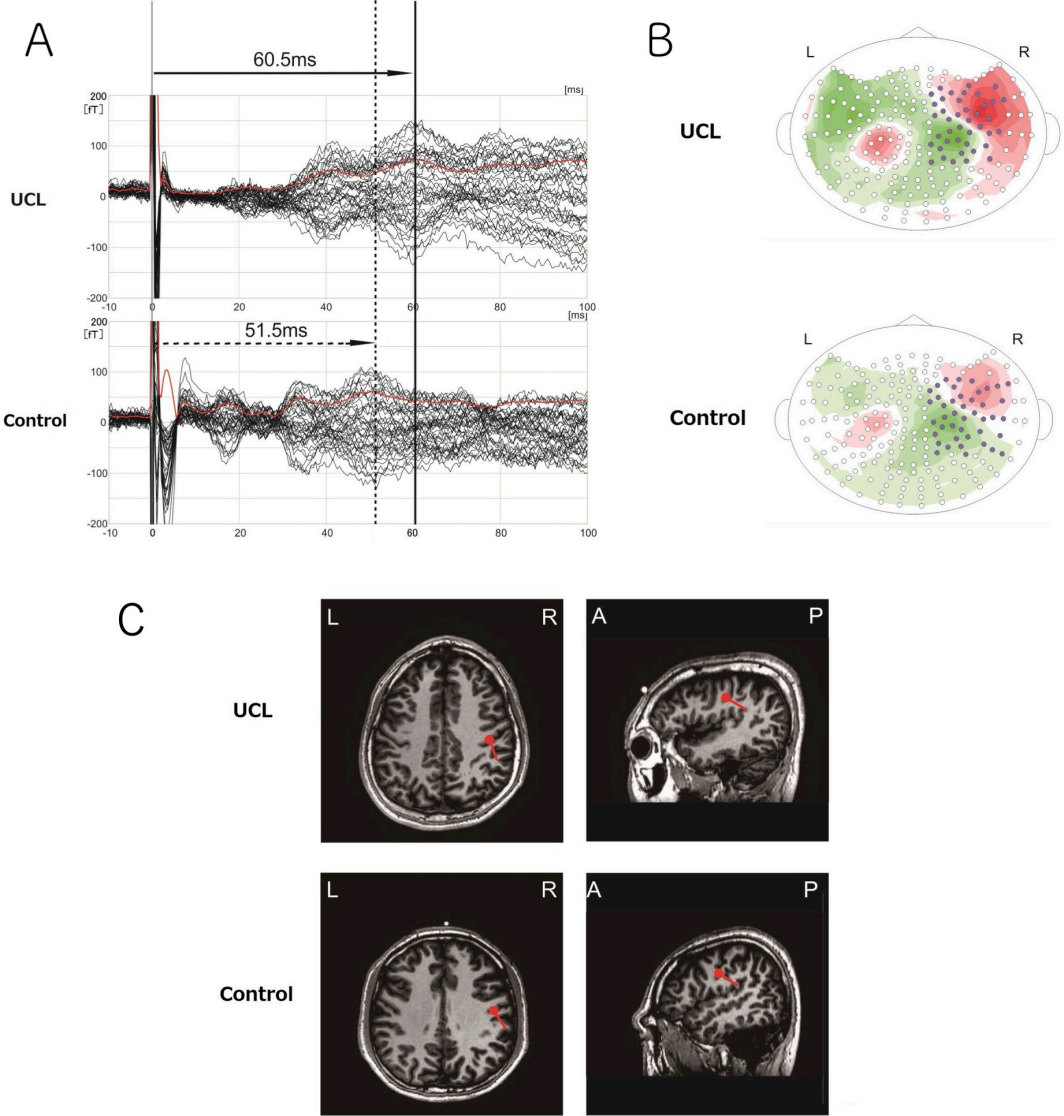
Reactions of the right hemisphere to electrical stimulation on the left side of the upper lip. In each figure, the data shown above are for a representative patient with left-sided cleft lip and the data shown below are those for a healthy subject. (A) Waveforms. (B) Isofield maps of the cP60m. (C) Magnetic fields of the cP60m. SEFs were modeled as ECDs and were superimposed on the MR images or a spherical model. ECDs were located on the central sulcus.

Intensities of the cP60m (mean ± SD) in the UCL group were 15.774 ± 14.863 nA•m, 10.936 ± 5.332 nA•m, 15.723 ± 6.004 nA•m, 14.001 ± 3.989 nA•m, and 12.639 ± 5.047 nA•m, respectively, on the non-cleft side, center, cleft side, lower left side, and lower right side. Values in the control group were 18.431 ± 9.993 nA•m, 14.241 ± 6.071 nA•m, 17.826 ± 7.409 nA•m, and 19.263 ± 8.616 nA•m, respectively, on the upper left and right sides (combined), center, lower left side, and lower right side. Signal intensity of the cP60m showed no significant difference between the UCL group and the control group (Fig 4). Table 4 presents results obtained for intensity of SEFs using multiple linear regression analysis. Fig 5 shows the waveforms (Fig 5A), isofield maps (Fig 5B), and ECD locations (Fig 5C) of a representative patient in the UCL group (left CL) and a healthy subject in the control group evoked by electrical stimuli applied to the left side of the upper lip.

**Table 4:** Results obtained for intensity of SEFs using multiple linear regression analysis.

	Coefficient	95% conf. interval		*p*-value
U-non-cleft side	-4.068	-13.283	5.146	0.380
U-center	-0.126	-4.361	4.109	0.952
U-cleft side	-2.526	-13.202	8.150	0.636
L-left side	-4.710	-12.869	3.448	0.246
L-right side	-4.940	-13.808	3.927	0.263

Age and sex adjusted. U, upper; L, lower.

## Discussion

### Tactile stimulation threshold

Tactile stimulation thresholds showed no significant difference between those of the UCL group and the control group, which is in agreement with earlier reports describing that patients with CL/P have no sensory abnormality [14, 15]. Nerve regeneration, collateral reinnervation, and central amplification might be involved in the recovery mechanism of impaired sensation of the upper lip in patients with CL after cheiloplasty, as discussed later.

In contrast, patients with CL/P have abnormal sensations of dynamic touch with cotton chips [16]. Fast adapting receptors respond to vibration and dynamic touch, whereas slow adapting receptors respond to static touch [37]. This difference in the sensory receptors might partly account for the inconsistent results.

### Latency of SEFs

Latencies of the cP60m were significantly longer on the non-cleft side, center, and the cleft side of the upper lip in the UCL group than in the control group (*p* < 0.05), in accordance with hypothesis #1 formed for this study. Giblin reported that a delay in sensory nerve conduction is reflected in the peak latency of somatosensory evoked potentials (SEPs) [38]. Ghali et al. reported that longer latency was observed in the SEPs of the mental nerve region after extraction of the third molar [39]. Sedden reported that the latency of SEPs is apparently prolonged by the effects of injury to nerve fibers on signals sent to the central nervous system. Peripheral nerve injuries are classified into three categories of neurapraxia, axonotmesis, and neurotmesis [40]. These injuries involve Wallerian degeneration (degeneration of the axon distal to nerve division) and misdirection (regeneration in the Schwann tube of another nerve) [40]. Degeneration or loss of the myelin sheath results in prolonged conduction time because conduction of excitation is delayed or stopped at the myelin sheath (segmental demyelination) [40]. Consequently, we infer that damage to the peripheral nerve is likely to affect its pathway and that conduction is delayed. The longer latency of SEFs observed for the lip on the non-cleft side might result from the effects of surgery on the neurotransmission pathway in the overall upper lip.

Furthermore, electrical stimulation used for this study stimulated all of Aβ, Aδ, and C fibers. Earlier studies using MEG and EEG reported that unmyelinated C fibers have much lower conduction velocity than those of myelinated Aβ or Aδ fibers [41–43]. It is possible that the latency was delayed because of the different conduction velocities of nerve fibers.

Table 1 shows the surgery that UCL group patients received. Considering the maxillary nerve anatomy of [44], we infer that neither palatoplasty, alveolar bone grafting, nor orthodontic treatment affects the upper and lower lip sensations. Although orthognathic surgery and lip revision surgery might affect lip sensory functions, the patients who received such surgery showed no significant difference compared to patients who received no surgery. One earlier report has described altered sensation after mandibular osteotomy [45], but another described that sensory capabilities of the upper lip had recovered completely by 3 months after surgery in the maxilla [46]. Moreover, Essik reported no significant difference in lip sensation after lip revision surgery between patients who had received lip revision surgery and those who had not [47]. Therefore, we inferred that it is unlikely that these surgeries affect upper lip sensation.

### Intensity of SEFs

This study found no significant difference in the signal intensity of SEFs between the UCL group and the control group, which finding is contrary to hypothesis #2. Differences in signal intensity in the facial region are related to the peripheral nerve density [27]. Comparison of lip tissue sections revealed no difference in the amount of nerve fibers between patients with CL before cheiloplasty and healthy babies aged 2–5 months after birth [48]. However, hypertrophic scarring caused by chronic inflammation and fibrosis persists for a long time after cheiloplasty [49, 50]. Nerve regeneration can be expected to be insufficient, depending on the scar size [51, 52]. Results suggest that the peripheral nerve density might have decreased at the surgical site because of scarring.

However, collateral sprouting is well known to occur from surrounding healthy tissues at sites of injury and denervation [53, 54]. Sensory collateral re-innervation in the facial region has been demonstrated by electrophysiological examination in a case of a major nerve removal [55]. Therefore, similar collateral sprouting could have occurred in our patients with CL from neighboring healthy nerves such as the infraorbital extranasal branches and/or zygomatic nerve facial branches. Furthermore, a study examining finger transplantation found that evaluation of the extent of nerve restoration using SEFs detected responses in the primary somatosensory cortex despite the absence of subjective sensation. The responses were found to be gradually approximating the response of the adjacent finger [56]. Decreased sensations in the periphery might be compensated by amplification in the center along with re-innervation. Furthermore, the signal intensity induced by lip stimulation in elderly people is higher because of central amplification [33]. Therefore, some central compensation mechanism might also account for the present findings of SEF intensity. However, additional research must be conducted to investigate these possibilities.

The limitations of this study are (1) the incomparability of surgical procedures, (2) the uncertainties of sensory impairment in patients with CL before cheiloplasty, and (3) the fact that the result was obtained with fewer than the required number of patients because of the study period and budget. Although evaluating lip sensation in infant patients with CL before cheiloplasty might present a difficult challenge for us, we would also like to evaluate the effects of CL and surgical techniques on lip sensation in future studies.

## Conclusions

Latencies of cP60m were significantly longer on the upper lip in the UCL group than in the control group, which indicates agreement with hypothesis #1. This study revealed no significant difference in the intensity of SEFs between groups, which is contrary to hypothesis #2.

Evaluation by SEFs revealed that somatic sensations in the upper lip patients with CL differed from those of healthy people. The results presented herein suggest that SEFs are useful for the objective evaluation of lip sensations. The same approach is concurrently under investigation to evaluate palatal sensations in patients with cleft palate. Establishing a new objective evaluation method using SEFs for somatic oral sensations might improve future surgery and oral functions of patients with CL/P.

## Supporting information

S1 File The values used to build graphs. (XLSX)
